# A pilot study of stereotactic boost for malignant epidural spinal cord compression: clinical significance and initial dosimetric evaluation

**DOI:** 10.1186/s13014-020-01710-4

**Published:** 2020-11-18

**Authors:** Elysia K. Donovan, Jeffrey Greenspoon, Kara L. Schnarr, Timothy J. Whelan, James R. Wright, Crystal Hann, Anthony Whitton, Tom Chow, Sameer Parpia, Anand Swaminath

**Affiliations:** 1 Present Address: Sunnybrook Odette Cancer Centre, Department of Radiation Oncology, University of Toronto, 2075 Bayview Avenue, T2 Wing, Toronto, ON M4N3M5 Canada; 2 Juravinski Cancer Centre, Department of Radiation Oncology, McMaster University, 3rd Floor, 699 Concession Street, Hamilton, ON L8V 5C2 Canada; 3 Juravinski Cancer Centre, Department of Medical Physics, McMaster University, 4th Floor, 699 Concession Street, Hamilton, ON L8V 5C2 Canada; 4 Juravinski Cancer Centre, Department of Oncology, McMaster University, 1st Floor, 699 Concession Street, Hamilton, ON L8V 5C2 Canada

**Keywords:** Spinal cord compression, Palliative radiation, Stereotactic radiotherapy

## Abstract

**Purpose:**

Metastatic epidural spinal cord compression (MESCC) is a devastating complication of advanced malignancy, which can result in neurologic complications and significant deterioration in overall function and quality of life. Most patients are not candidates for optimal surgical decompression and as a result, receive urgent 3D conformal radiotherapy (3DCRT) to prevent or attempt to reverse neurologic progression. Multiple trials indicate that response and ambulatory rates after 3DCRT are inferior to surgery. The advent of stereotactic body radiation therapy (SBRT) has created a method with which a “radiosurgical decompression” boost may facilitate improve outcomes for MESCC patients.

**Methods:**

We are conducting a pilot study to investigate SBRT boost after urgent 3D CRT for patients with MESCC. The aim of the study is to establish feasibility of this two-phase treatment regimen, and secondarily to characterize post-treatment ambulation status, motor response, pain control, quality of life and survival.

**Discussion:**

We describe the study protocol and present a case report of one patient. A quality assurance review was conducted after the first seven patients, and resultant dose-constraints were revised to improve safety and feasibility of planning through more conservative organ at risk constraints. There have been no severe adverse events (grade 3–5) to date. We have illustrated clinical and dosimetric data of an example case, where a patient regained full strength and ambulatory capacity.

**Conclusions:**

Our study aims to determine if SBRT is a feasible option in addition to standard 3DCRT for MESCC patients, with the goal to consider future randomized trials if successful. Having a robust quality assurance process in this study ensures translatability going forward if future trials with multicenter and increased patient representation are to be considered.

**Trial registration:**

clinicaltrials.gov; registration no. NCT03529708; https://clinicaltrials.gov/ct2/show/NCT03529708; First posted May 18, 2018.

## Introduction

Metastatic epidural spinal cord compression (MESCC) is a devastating complication of advanced malignancy which can result in a significant deterioration in physical function and quality of life (QoL). Patients typically present with both radiographic evidence and clinical symptoms of motor and sensory deficits, as well as bowel and bladder dysfunction [1]. Treatment is pursued urgently, with initial surgical decompression considered the gold standard with respect to maximizing the chances of neurologic preservation or recovery in surgical candidates [2]. Surgical candidacy is contingent on other common factors including controlled systemic disease, single-level vertebral disease, and appropriate performance and motor function. Thus only a minority of patients, (less than 15%) are typically eligible for surgical decompression.^3^

A majority of patients are therefore generally treated with less aggressive therapies, most commonly 3D conformal palliative radiotherapy (3DCRT) alone, delivered in one to ten fractions [1–4]. Studies have indicated that regardless of 3DCRT fractionation regimen, overall response rates and post-treatment ambulatory rates are approximately 70%. This is in comparison to much higher post-surgical ambulatory rates at approximately 85% [1–6]. Furthermore, the durability of radiotherapy (RT) response, requirement of re-treatment, and effect on quality of life (QoL) remain pertinent issues requiring optimization [7]. With the development of new technologies, including high-dose ablative stereotactic body radiation therapy (SBRT) techniques, there may be opportunities to optimize response rates and durability of treatment for MESCC [8, 9].

Our institution is currently conducting a pilot trial to investigate the potential benefits of a sequential RT “boost” dose of delivered through the use of SBRT following standard urgent conventional external beam (3DCRT). This approach facilitates necessary and urgent standard up-front conventional 3DCRT for MESCC, while simultaneously creating the opportunity for dose escalation using an SBRT boost following 3D-CRT. The primary objective of the study is to assess the ability to effectively recruit, plan and deliver a boost dose using SBRT after standard 3DCRT, however we also aim to characterize whether improvements are achieved with respect to motor and ambulatory status as well as tumor control.

## Methods

### Study design

We are conducting a single arm, prospectively designed pilot trial of SBRT boost following 3DCRT for MESCC. With a planned sample size of 30 patients, we estimate the proportion of patients that will be successfully planned and treated with SBRT boost (whilst achieving both target and normal tissue objectives) within 6 weeks of 3DCRT will be 80%, to within a 95% confidence interval of 75–97%. This study has been approved by the local Institutional Research Ethics Board (REB).

### Patient selection

Patients with radiologic and/or clinical MESCC are recruited from a tertiary academic Radiation Oncology center. Eligible patients have a diagnosis of non-hematologic metastatic malignancy, with MESCC defined by presence of epidural tumor with evidence of contact with spinal cord on CT or MRI, and a motor score of 3 or greater on the Rades Motor Function Scale (defined by movement against gravity) [6]. Patients who are deemed surgical candidates, who have instability of vertebral bodies, or who had previous in-field radiotherapy are excluded. Recruitment was initiated in November 2018 and is ongoing.

### Phase I simulation and planning guidelines (3DCRT)

Patients on this study are initially prescribed either 8 Gy in 1 fraction or 20 Gy in 5 fractions for 3DCRT, delivered urgently by a parallel opposed pair or direct beam arrangement. Treatment volume, energy, dose and technique are at the clinician’s discretion, and are meant to facilitate same-day planning and treatment as per usual standard of care practice.

### Phase II simulation (SBRT boost)

SBRT simulation imaging includes both spine MRI with gadolinium and CT data scans. Images are then exported and fused in the Accuray Cyberknife Multiplan^®^ planning system (Sunnyvale, CA). A gross tumor volume (GTV) is created to encompass any tumor in the vertebral body and canal as defined on Magnetic Resonance Imaging (MRI) T1 post-gadolinium sequences. The clinical target volume (CTV) includes the GTV plus the entire vertebral body on CT, and an isotropic margin of 2 mm is added to create the planning target volume (PTV).

The planned prescription dose is 24 Gy in 2 fractions prescribed to the PTV based on the initial SC.24 study interventional arm dose [14]; however, to meet organ at risk (OAR) constraints the prescription dose may be reduced to as low as 18 Gy. Patients therefore receive between 18 Gy-24 Gy prescription dose for phase II of the treatment. At least 70% of the PTV must be covered by the prescription dose. Editing of GTV and CTV is allowed at the discretion of the radiation oncologist in the case where disease volume is deemed to be too large to be entirely encompassed. The rationale for this is to achieve coverage at the region where gross tumor decompression from the spinal cord is deemed necessary.

Phase II is delivered 4–6 weeks after phase I treatment. Patients are required to hold chemotherapy, targeted therapy, or immunotherapy seven days prior to and following the SBRT boost. Patients may continue anti-hormonal therapy while receiving treatment if applicable. The time period of 4–6 weeks for delivery of phase II after phase I allows for compatibility with systemic therapy schedules (i.e. to have SBRT coincide with treatment breaks to minimize time off systemic therapy). Patients are monitored for toxicity and QoL at least once on-treatment, and then at 4 weeks, 3 months and 6 months following treatment. Assessments include motor response (according to Rades et al. [6]), ambulatory and pain (ten point visual analog) scales, toxicity (using Common Terminology Criteria for Adverse Events V4.03) [10], QoL using the European Organization for Research and Treatment of Cancer Quality of Life Questionnaire core-30 (EORTC QLQ-C30) and Bone-Metastasis 22 (EORTC BM-22) [11, 12], overall survival, and local control using MRI at 3 and 6 months following the Phase II boost.

### Initial dosimetry of combined phase I and II plans

Composite plans of phase I and phase II were initially created by importing both plans into MIM (Beachwood, OH), and calculating combined accumulated dose to both targets and OARs. The initial dose constraint criteria for the study are presented in Table 1. The constraint for each OAR indicates the maximum point or mean dose allowed for each critical structure on a composite plan of phase I and phase II. This constraint table was based on previous OAR constraints suggested in the literature and studies of spine re-irradiation [13–15].

**Table 1:** Planning constraints for (a) initial composite plan of phase I (3DCRT; 8 Gy/1 fraction or 20 Gy/5 fractions) and phase II (2-fraction SBRT Cyberknife boost) treatment; and (b) revised phase II SBRT Cyberknife boost plan only

Target/OAR	Parameter	Initial composite plan		Revised phase II only	
		3-fraction combined	7-fraction combined	Phase I Dose 8 Gy/1 fraction	Phase I Dose 20 Gy/5 fractions
Spinal cord	Maximum PRV Point Dose	23 Gy	32 Gy	15 Gy	12 Gy
Sacral nerve roots	Maximum point dose	26 Gy	32 Gy	26 Gy	32 Gy
Kidneys	Mean Whole Kidney Dose	6 Gy	6 Gy	6 Gy	6 Gy
	Maximum point dose	26 Gy	26 Gy	26 Gy	26 Gy
Esophagus	Maximum point dose	25.5 Gy	35 Gy	20 Gy	15 Gy
Small Bowel	Maximum point dose	27 Gy	32 Gy	20 Gy	15 Gy
Stomach	Maximum point dose	30 Gy	35 Gy	20 Gy	15 Gy
Large Bowel	Maximum point dose	34.5 Gy	40 Gy	20 Gy	15 Gy
Trachea	Maximum point dose	30 Gy	40 Gy	20 Gy	15 Gy
Liver	Mean whole liver	8–9 Gy	8–9 Gy	8 Gy	8 Gy
	Dose per volume	D700cc < 17.1 Gy	D700cc < 21 Gy	D700cc < 17 Gy	D700cc < 21 Gy
Lung	Dose per Volume	V11Gy < 37%	V13.5 Gy < 37%	V11 Gy < 37%	V13.5 Gy < 37%

*PRV *planning risk volume, *Gy *gray

## Results

### Clinical case

**Fig. 1:**
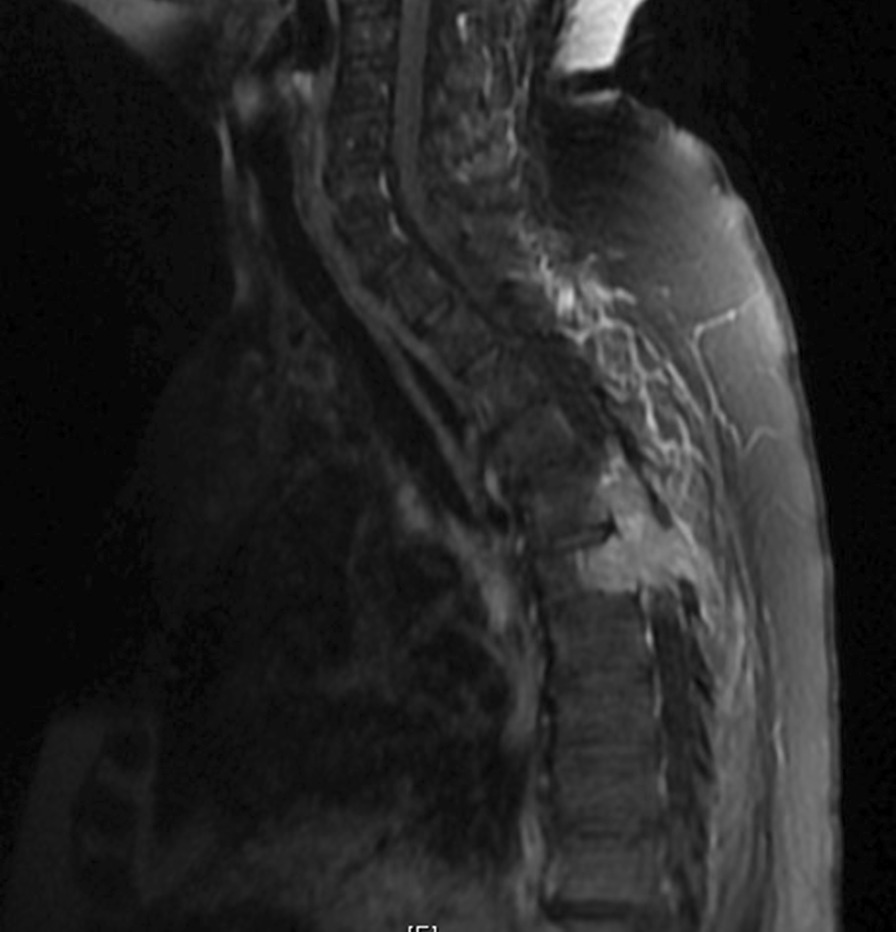
Baseline T1 (left) MRI sequences showing soft tissue metastasis at T6 with epidural soft tissue component and edema and extension to spinal cord in Patient A

A 62 female with history of breast cancer (referred to herein as “Patient A”) presented with bilateral lower extremity weakness and paresthesia over 2 weeks, 7 of 10 back mid-back pain, and inability to ambulate for 2 days. On exam Patient A had ability to flex hip and extend and flex knee against gravity but not resistance (score of 3 out of 7 on the Rades motor function scale). MRI indicated a large soft tissue mass at the T6 vertebral body with spinal canal effacement and cord compression (Fig. 1).

**Fig. 2:**
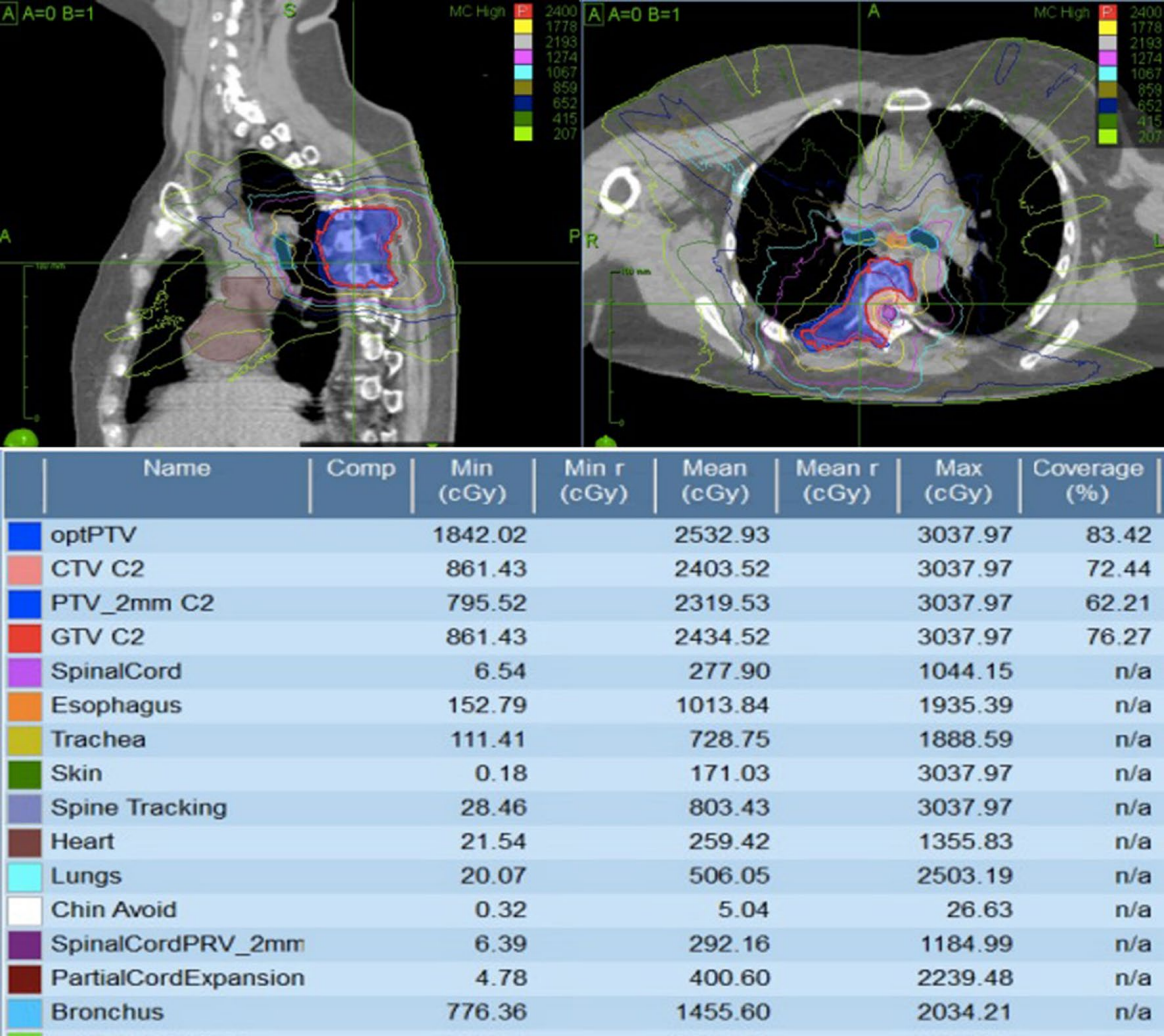
Phase II Cyberknife plan for Patient A, Isodose distribution (topand radiotherapy target and organ at risk doses (bottom) are displayed

She received 20 Gy in 5 fractions encompassing T4–7 using a posterior direct 10MV beam prescribed to 5 cm for phase I treatment. The maximum dose to the spinal cord was approximately 19.8 Gy, the maximum dose to the esophagus was 17.5 Gy and the entire CTV was covered by approximately 18 Gy in phase I. Four weeks later she received the phase II boost (Fig. 2), with 24 Gy in 2 fractions prescribed to the PTV. Eighty four percent (83%) of the CTV received 24 Gy in phase II, with the spinal cord planning risk volume (PRV) receiving 11.85 Gy maximum point dose, and the esophagus receiving 19 Gy maximum point dose.

**Fig. 3:**
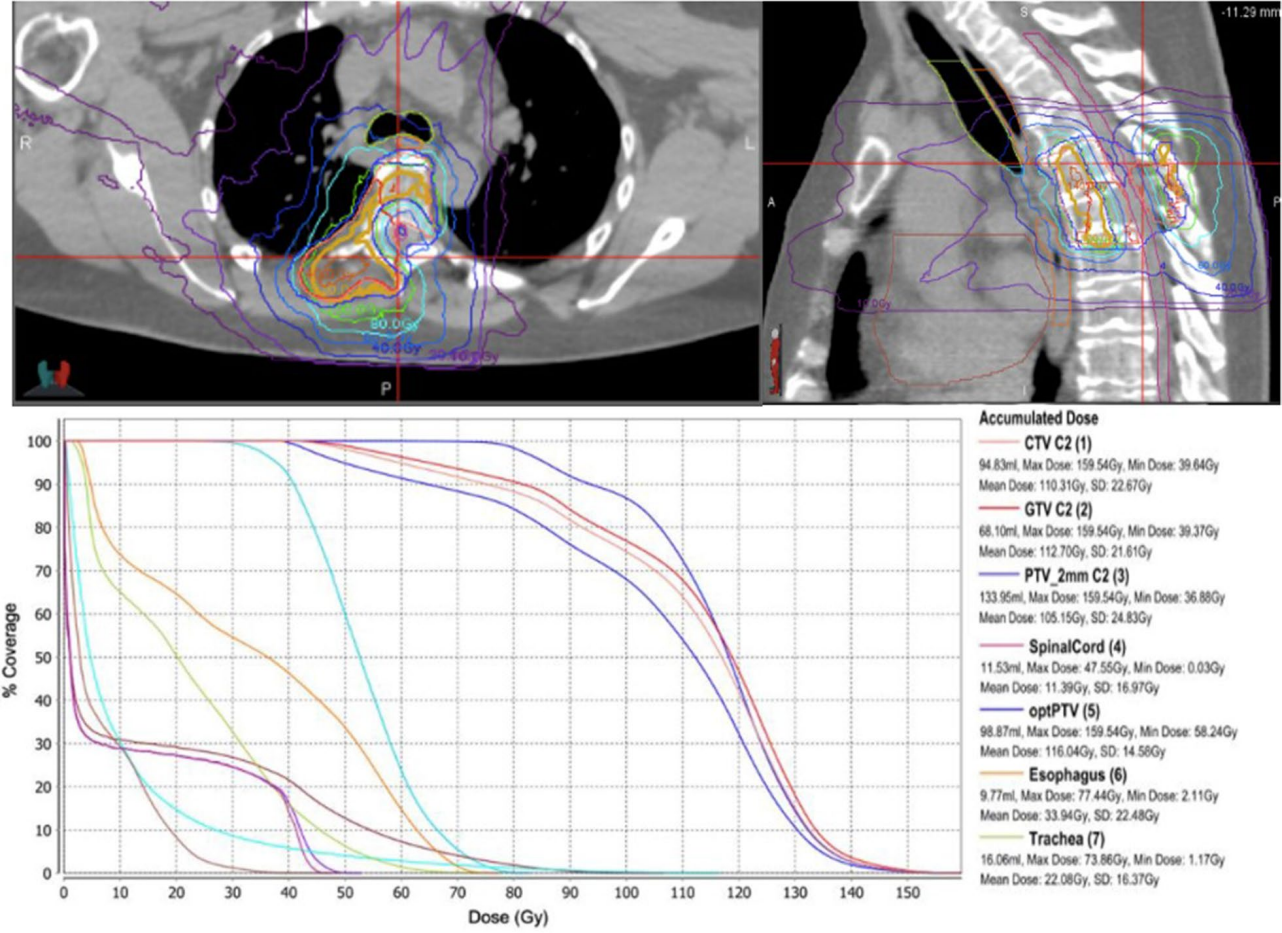
Composite plan for Patient A. Composite distribution (top), dose volume histogram and accumulated estimated phase I and II doses (bottom) displayed

On the composite plan displayed in Fig. 3, however, the maximum dose to the spinal cord was reported as 47 Gy, which was a significant overestimate of the combined dose receiving in phase I and phase II (summated as approximately 31 Gy over 7 fractions). Similarly, the esophagus maximum dose was reported as 75 Gy while the esophagus was reported to have received approximately 36 Gy over both courses. Overall the composite plan indicated that 95% of the CTV received 55 Gy, which is also an overestimate of the dose received to the target. Figures 2 and 3 show both the SBRT boost plan, and composite plan of phase I and phase II respectively. This example illustrates the challenges with using a composite plan in the delivery of an SBRT boost, and the impetus for revision of dose constraints for a more accurate assessment of the phase II plan only.

**Fig. 4:**
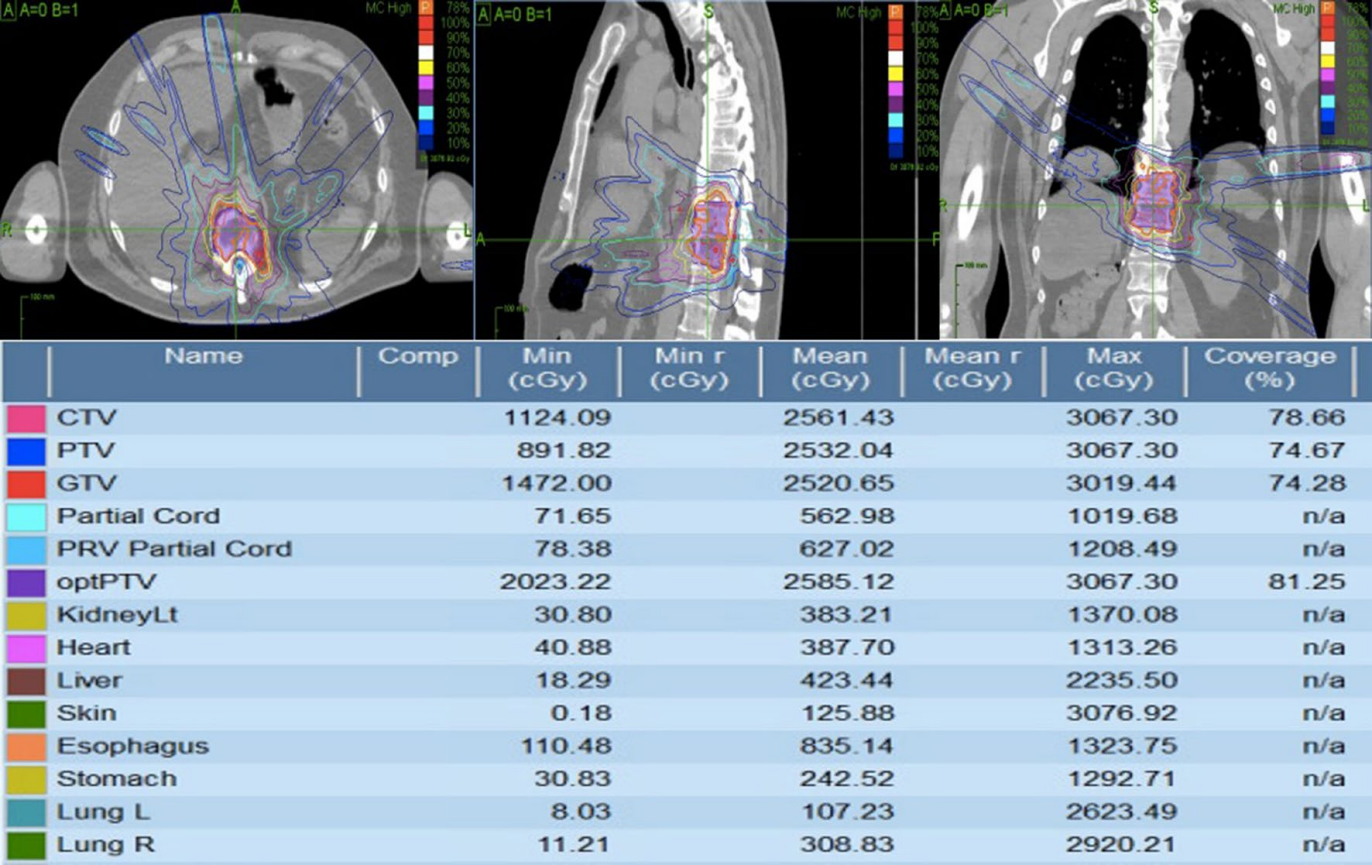
Phase II Cyberknife plan for Patient B treated after revision of dose constraints. Phase II distribution (top) and radiotherapy target/organ at risk doses (bottom) are displayed

Three months post-treatment Patient A presented for follow up reporting 1 out of 10 pain in the mid-back, and after two months of physiotherapy was again ambulating with a walker. All muscles of the lower extremity were functioning at full strength on examination (7 of 7 Rades score). MRI indicated regression of T6 soft tissue metastasis with complete regression of tumor from the spinal canal (Fig. 4). No significant neurological or gastrointestinal toxicity was observed.

### Quality assurance and revision of phase II plan constraints

In this study, we conducted a quality assurance (QA) process involving peer review by two radiation oncologists and a medical physicist prior to delivery of phase II of treatment. After the first seven patients, the study protocol was reassessed through this process (QA). Challenges were recognized in the workflow and efficiency of creating the composite plan in a timely manner prior to the required time for phase II plan development, QA, and treatment delivery. The accuracy of the composite plan (an estimate of the phase I and II plans superimposed on the patient’s anatomy on phase II simulation data scan) was also noted as being limited by variations in patient’s anatomy and positioning.

As a result, more conservative dose constraints were adapted, and the planning guidelines were revised to reflect the OAR limits for phase II only. This negates the need for mandatory creation of a composite plan prior to delivering phase II, while reducing the risk of toxicity through more conservative planning constraints (composite plans are now created post Phase II delivery for subsequent analysis). The revised OAR constraints (Table 1) therefore assume that the spinal cord receives the maximum dose delivered in phase I. Phase I plans are still reviewed prior to phase II planning to ensure the maximum assumed dose is not exceeded.

**Fig. 5:**
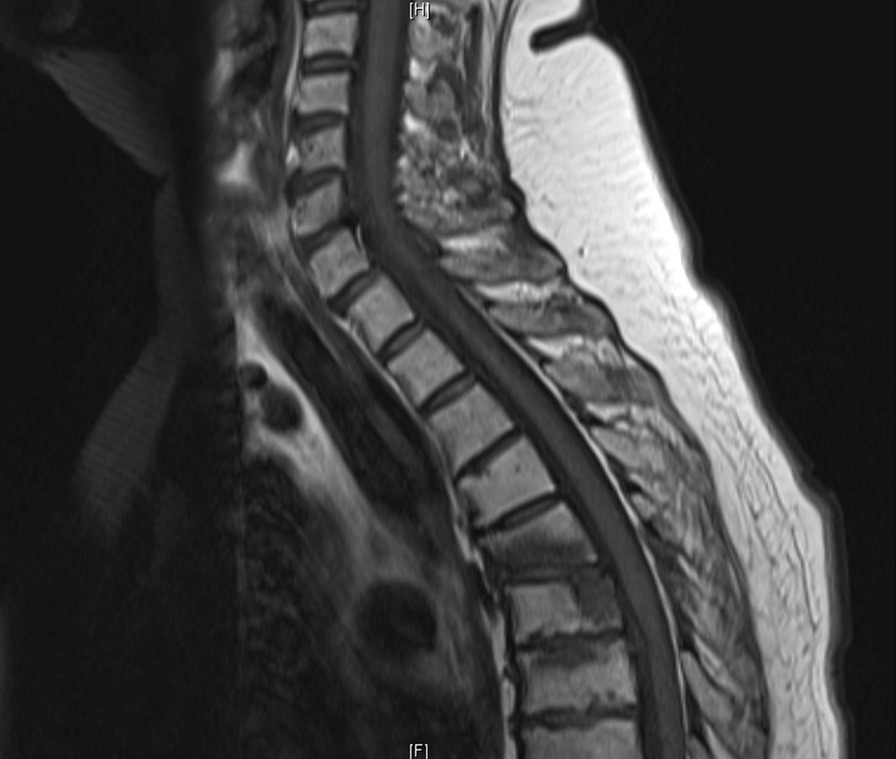
T2 (right) MRI sequence at 3 months showing excellent response to phase II boost treatment in Patient A

See below for examples. Patient A was treated using the composite approach (Fig. 3), while Fig. 5 demonstrates a phase II plan of “Patient B” treated with revised dose constraints (as per Table 1) following the QA process.

## Discussion

### Clinical significance

A recent systematic review of 3DCRT for MESCC indicated that despite no clear difference in motor outcomes or survival between single and short course regimens, patients still achieve inferior outcomes compared with surgical patients in motor response and ambulation, durability of response, and survival with 3DCRT [4]. Approximately 30% of patients required re-treatment of the spinal cord after 3DCRT, and a low proportion of patients were able to regain ambulatory capacity if initially non-ambulatory, indicating that there may be patients who will benefit from dose-escalated RT [4]. Inferior outcomes have also been demonstrating in MESCC receiving longer courses of radiotherapy [6]. Furthermore, while QoL is a central issue for patients with MESCC, studies suggest a decline in QoL over time post-radiotherapy in contrast to a steady increase post surgery [7, 8].

This highlights the opportunity for further investigation into optimal treatment for MESCC, and potential opportunities for advancement with the evolution of RT technology [14–16]. The advent of SBRT has already provided a possible mechanism to achieve outcomes comparable to surgical treatment in other anatomic sites such as lung and brain [17, 18]. Furthermore, while it has been used effectively in the setting of spinal bone metastasis, and re-irradiation of spinal tumors, SBRT has not been thoroughly investigated in the setting of upfront MESCC [14–16, 19–23].

Patients on this trial are initially treated urgently with palliative 3DCRT, and then at 4–6 weeks later with an SBRT boost. The rationale for allowing a range of treatment time from 4 to 6 weeks is to minimize treatment breaks from targeted or cytotoxic systemic therapy regimens (for example, if a patient has a natural chemotherapy break at week 5, SBRT boost can be administered during this time). Therefore, the entire treatment is completed within 6 weeks of diagnosis of MESCC. Previous studies have indicated that a longer overall treatment time for short course 3DCRT is not associated with either post-treatment motor function or survival [24]; however the effect of waiting 4–6 weeks for the SBRT boost is unclear. Nonetheless, we felt this was a good trade-off of risk of toxicity with an SBRT boost too close to 3DCRT versus a longer wait which may not result in a high chance of successful recruitment of patients given the poor prognosis of MESCC in general.^5^

Investigators have previously reported superior pain control using SBRT alone in patients with vertebral metastasis but no epidural tumor or neurologic symptoms approaching 90%, and local control of over 80% [16]. Ryu et al. reported epidural tumor volume reduction of 65% and thecal sac patency improvement from 55 to 75%, two months post SBRT monotherapy in patients who presented with tumor in the spinal cord on MRI. In this study, 94% of 36 neurologically intact patients at presentation retained their status, while 52% of patients presenting with pain, motor or sensory deficit recovered to normal [19]. Re-treatment with SBRT following recurrence of irradiated spinal lesions has also been used safely in many studies, with fracture rates and myelopathy as low as 3–12% and 1.2% respectively [14, 20–23]. A recent systematic review reports 1 year LC of 76%, and improvement in pain scores ranging from 65 to 81% in patients with spine re-irradiation using SBRT [21]. These experiences have confirmed that dose escalation to the spine in this context is tolerable and safe, yet the optimal treatment regimen for MESCC patients is still unknown.

Randomized trials such as CCTG SC.24 are attempting to evaluate the role of SBRT alone (24 Gy in 2 fractions) in comparison to standard palliative RT (20 Gy in 5 fractions) for patients with vertebral metastases without MESCC in two fractions [14]. The patient population however, includes those with minimal symptoms, without neurologic compromise and good performance status, and is not necessarily representative of many patients with MESCC. MESCC often results in complex pain, decreased functional and ambulatory ability, and it is currently unknown whether dose-escalated therapy may serve to better reverse neurologic impairment for patients than standard treatments. Challenges also exist in the compatibility of delivering RT urgently for MESCC with time required for resource intensive simulation, planning and delivery process for SBRT. This could potentially further compromise neurologic function in already symptomatic MESCC patients. On the other hand, suboptimal 3DCRT doses could also limit tumor control, ambulatory status and durability of pain relief in MESCC.

Despite limited safety demonstrated by spinal SBRT in re-irradiation studies, there is little evidence to evaluate the additional benefit and risk of SBRT in the context of a “radiosurgical” decompression boost treatment following conventional RT (similar to the effect of upfront surgery plus radiation) [2]. Benefits could include improved motor and ambulatory status, and reduced requirements for re-treatment. This approach has the potential to serve as an intermediary between radical spinal SBRT alone and urgent palliative RT, accomplishing both rapid treatment and dose escalation up-front. Established normal tissue tolerances, based on re-irradiation data in spine SBRT, can still be respected with careful SBRT planning [14, 21, 23]. For MESCC patients, an SBRT boost has the potential to exceed traditional outcomes, with the additional benefits of limited invasiveness and convenience.

### Quality assurance

A robust QA process is particularly important in the setting of high dose per fraction radiotherapy, where small differences in actual versus expected dose can result in significant toxicities to normal tissue. This has been emphasized in previous studies involving hypofractionation [25]. Consistent review is therefore considered invaluable in this trial and resulted in substantial changes to the workflow process thus far. For example, composite plans are often created to estimate the radiotherapy doses to both targets and normal structures of two separate treatment courses. This can be challenging, however, when different positioning and immobilization are used. We identified early on that while the position of the spinal bones is presumed to be similar between scans, movement in other OARs made it difficult to estimate total dose received. For example both stomach and small bowel are mobile within the patient, and differences in position can occur between the two phases of treatment and between fractions. This is particularly relevant in the case of SBRT, as both of these organs are highly sensitive to radiotherapy and even small differences in position can dramatically alter delivered dose. Additionally, in order to capture patients receiving urgent radiotherapy for spinal cord compression, CT simulation is performed on an urgent basis. The high-resolution MR-simulation required to delineate the tumor volume and spinal cord for the SBRT boost is therefore done at a later date, at which point positioning and anatomy may have changed.

Through the QA process, a decision was made to amend OAR dose constraints to be more conservative and assume that each organ received the maximum dose during the first course. The second SBRT boost course is now assessed based on these constraints prior to plan approval. This also ultimately allowed for greater efficiency in workflow and patient safety. This quality assurance process will be continued through the duration of the trial in a similar manner to optimize patient safety and technical efficiency.

There are a number of limitations to our study, which must be considered when interpreting results. This is a phase I pilot study, including only a small number of patients who met strict inclusion criteria. For example, patients must have at least some degree of power in legs against gravity (Rades score of 3), and must have stable vertebral bodies. Outcomes therefore cannot be applied to patients with very poor prognosis, and lacking power in the legs, or with unstable vertebrae. Further, given there is no comparison arm or randomization in this study, patient outcomes must ultimately be compared to historical studies, where patients may have different baseline characteristics and motor status. Finally, the introduction of steroids and pain medication during treatment course may also affect motor, ambulatory and quality of life outcomes. It is difficult to standardize medication regimens however due to differential clinical needs of this patient population. Given the potential sources of bias discussed, results must be interpreted cautiously, and further studies need to be conducted prior to routine implementation of SBRT boost for MESCC.

## Conclusion

Ultimately, our study aims to determine whether the regimen of 3DCRT plus SBRT boost is feasible in the MESCC patient population, and whether a potential benefit exists compared with previous studies with respect to motor response (40%) and ambulation (70%). If this feasibility study is successful, a randomized study of SBRT boost compared with 3DCRT alone could be considered. As ambulation rate is intimately associated with overall survival, motor response and ambulation are extremely important outcomes in these patients [26, 27]. Our study has the potential to make a significant clinical impact by assessing whether SBRT boost should be routinely considered for MESCC patients. Furthermore, a need to refine our QA processes early on this trial was of utmost importance, particularly if considering future studies that capture larger numbers of patients and involve multicenter representation. While the results thus far are promising, we await final study results prior to further research or clinical implementation.

## Data Availability

Research data are not available currently, as this is an ongoing trial.

## Abbreviations

3DCRT: 3 Dimensional conformal radiotherapy
CCTG: Canadian Clinical Trials Group
CTV: Clinical target volume
GTV: Gross tumor volume
MESCC: Malignant epidural spinal cord compression
OAR: Organ at risk
PRV: Planning risk volume
PTV: Planning target volume
QA: Quality assurance
QoL: Quality of life
RT: Radiotherapy
SBRT: Stereotactic body radiation therapy
